# Aetiological Understanding of Fibromyalgia, Irritable Bowel Syndrome, Chronic Fatigue Syndrome and Classificatory Analogues: A Systematic Umbrella Review

**DOI:** 10.32872/cpe.11179

**Published:** 2023-09-29

**Authors:** Maria Kleinstäuber, Andreas Schröder, Sarah Daehler, Karen Johanne Pallesen, Charlotte U. Rask, Mathias Sanyer, Omer Van den Bergh, Marie Weinreich Petersen, Judith G. M. Rosmalen

**Affiliations:** 1 Department of Psychology, Emma Eccles Jones College of Education and Human Services, Utah State University, Logan, UT, USA; 2 The Research Clinic for Functional Disorders and Psychosomatics, Aarhus University Hospital, Aarhus, Denmark; 3 Danish Centre for Mindfulness, Aarhus University, Aarhus, Denmark; 4 Department of Child and Adolescent Psychiatry, Psychiatry, Aarhus University Hospital, Aarhus, Denmark; 5 Department of Health Psychology, University of Leuven, Leuven, Belgium; 6 University Medical Centre Groningen, University of Groningen, Groningen, The Netherlands; Philipps-University of Marburg, Marburg, Germany

**Keywords:** chronic fatigue syndrome, myalgic encephalomyelitis, aetiology, fibromyalgia, irritable bowel syndrome, functional somatic syndromes, systematic review

## Abstract

**Background:**

This umbrella review systematically assesses the variety and relative dominance of current aetiological views within the scientific literature for the three most investigated symptom-defined functional somatic syndromes (FSS) and their classificatory analogues within psychiatry and psychology.

**Method:**

An umbrella review of narrative and systematic reviews with and without meta-analyses based on a search of electronic databases (PubMed, Web of Science, Embase, PsychINFO) was conducted. Eligible reviews were published in English, focused on research of any kind of aetiological factors in adults diagnosed with fibromyalgia syndrome (FMS), irritable bowel syndrome (IBS), chronic fatigue syndrome/myalgic encephalomyelitis (CFS/ME), and somatic symptom disorder (SSD)/somatoform disorder (SFD).

**Results:**

We included 452 reviews (132 systematic reviews including meta-analyses, 133 systematic reviews, 197 narrative reviews), of which 132 (29%) focused on two or more of the investigated health conditions simultaneously. Across diagnoses, biological factors were addressed in 90% (k = 405), psychological in 33% (k = 150), social in 12% (k = 54), and healthcare factors in 5% (k = 23) of the reviews. The methodological quality of the included systematic reviews (k = 255) was low (low/critically low: 41% [k = 104]; moderate: 49% [k = 126]; high quality: 10% [k = 25]). The high-quality systematic reviews suggest that deficient conditioned pain modulation, genetic factors, changes in the immune, endocrinological, gastrointestinal, cardiovascular, and nervous system, and psychosocial factors such as sexual abuse and pain catastrophizing increase the risk for FSS.

**Conclusion:**

Only very few systematic reviews have used comprehensive, biopsychosocial disease models to guide the selection of aetiological factors in FSS research. Future research should strive for higher scientific standards and broaden its perspective on these health conditions.

## Highlights

- This is an umbrella review of 452 reviews on the aetiology of FSS.
- Biological factors were most commonly examined as correlates of FSS.
- In 90% of the included reviews the methodological quality was low to moderate.
- High-quality reviews found genetic, immuno-endocrinologic, GI, and CV risk factors.
- High-quality reviews found psychosocial risk factors: sexual abuse, catastrophizing.

How physicians conceptualize disease determines their attitude towards their patients and the problems they present (Engel, 1977). This is particularly relevant in case of the so-called *functional somatic syndromes (FSS)* (Henningsen et al., 2007; Wessely et al., 1999). FSS are characterised by somatic symptoms that currently cannot be attributed to reproducibly observable pathophysiological processes, described by the rather outdated but in the past very popular term of *medically unexplained symptoms (MUS)*. Medicine has a long tradition of struggling with classifying and understanding FSS within the traditional disease model, resulting in a large variety of diagnostic labels that reflect the socio-cultural characteristics of a particular decade (neurasthenia, DaCosta syndrome, soldier’s heart syndrome, etc.) (Barsky & Borus, 1999). In recent decades, FSS have typically been investigated within a biopsychosocial model (Engel, 1977), but substantial differences exist between physicians in their belief about the relative importance of certain factors to understand these syndromes. Physicians who adhere to a purely biomedical model might consider these health problems as non-diseases, resulting in reduced scientific interest and neglect in patient care. In contrast, physicians’ overemphasis on psychosocial explanations might influence how such health problems are perceived publicly and might induce stigma.

Epidemiological research suggests that FSS are closely related and partly overlapping (Donnachie et al., 2020; Fink & Schröder, 2010; Janssens et al., 2015; Wessely et al., 1999), although some syndrome-specific aetiological factors have been found (Hamilton et al., 2009). Nevertheless, a variety of diagnostic labels are used, each based on the presence of a selected set of symptoms (Fink & Schröder, 2010), leading to unwanted diversity in diagnostic practice and clinical management (Budtz-Lilly et al., 2015; Creed, 2006; Wolfe, 2009). Diagnoses of FSS that are common in general medical settings as well as in medical specialties such as rheumatology, gastroenterology, or neurology are *fibromyalgia syndrome (FMS)* (Clauw, 2014), *irritable bowel syndrome (IBS)* (Ford et al., 2018), or *chronic fatigue syndrome/myalgic encephalomyelitis (CFS/ME)* (Haney et al., 2015). Whereas in psychiatry and in psychology the diagnostic label of somatoform disorders (SFD) was introduced in the 4^th^ edition of the Diagnostic and Statistical Manual for Mental Disorders (DSM-IV; American Psychiatric Association, 2000). The diagnostic category of SFD which mainly focused on excluding a medical explanation of the somatic symptom(s) was replaced by the *somatic symptom disorders (SSD)* in the 5^th^ edition of the DSM (DSM-5; American Psychiatric Association, 2013). SSD include syndromes of medically unexplained as well as syndromes of explained symptoms and – in comparison DSM-IV – rather emphasise the psychological distress associated with poor symptom management and psychological features such as extensive anxiety, dysfunctional thoughts and behaviours associated with the somatic symptom. Besides these diagnostic entities in DSM-IV and -5, several other concepts of MUS were established. For example in research literature concepts of multiple MUS such as the somatic symptom index-4/6 (Escobar et al., 1989) were introduced. In this paper we will refer to single FSS (including IBS, FMS, CFS/ME). We will summarise studies that include patients with multiple MUS in a sense of somatoform disorders, or other syndromes of multiple MUS under the umbrella term SSD.

This variety of diagnostic labels reflects also different aetiological views (Ford et al., 2018; Haney et al., 2015; Schröder & Fink, 2011) with important consequences. For example, compared to receiving a somatic diagnosis, a psychiatric diagnosis for FSS-related symptoms importantly impacts the patient’s behaviour, the patient-physician interaction (Budtz-Lilly et al., 2015; Wolfe, 2009), and is associated with more stigma. Previous research has shown that if patients present their symptoms with a more somatic versus psychosocial focus, they are more likely to receive a somatic diagnosis (Salmon et al., 2007). Consequently, receiving a psychiatric vs. somatic diagnosis for the same problem might influence the availability of certain healthcare services for patients.

FSS are highly prevalent, up to 22% in primary care (De Waal et al., 2004) and up to 66% in some medical specialties (Nimnuan et al., 2001). Associated functional limitations are as severe as in well-defined chronic physical diseases (Joustra et al., 2015). Direct medical costs and indirect costs as a consequence of sick leave and disability are high (Ford et al., 2018; Rask et al., 2015). Given this high prevalence and associated burden, a shared conceptualisation of FSS is urgently needed in order to optimise clinical management (Murray et al., 2016; Yon et al., 2015).

Facing this challenge, we systematically assessed the variety and relative dominance of current aetiological views – i.e., from genes to biochemistry, pathophysiology, individual psychological features, and cultural and healthcare factors – as represented in the scientific reviews on these health conditions across different syndrome definitions. We selected the three most well-described FSS (IBS, FMS, CFS/ME) and the somatic symptom disorders (SSD, focusing on individuals with syndromes of multiple medically unexplained symptoms or the precedingly used diagnostic label of somatoform disorders). The objectives of this review were to identify the predominant aetiological factors and proposed illness mechanisms in existing research literature to explain FSS and SSD, and to explore the level of evidence for aetiological factors and proposed illness mechanisms according to systematic reviews and meta-analyses. The methodological quality of reviews for specific investigated aetiological factors across syndrome definitions was evaluated, and the few currently well-documented aetiological factors are discussed. Finally, we provide implications for research.

## Method

### Literature Search

A literature search was performed in Medline (PubMed) and Embase (Embase.com) in January 2016, in PsycInfo (OVID) and Web of Science (Clarivate Analytics) in February 2016. The searches were updated in August 2017, February 2020, and January 2022. We included meta-analyses, systematic reviews, and narrative reviews, published in English between 1990 and the search dates, which focus on research of aetiological factors and/or illness mechanisms in functional somatic syndromes (FSS) and somatic symptom disorders (SSD) in adults. Narrative reviews were included to give a comprehensive overview as they form a large part of the available reviews. More specifically, we included reviews on the three most investigated FSS – chronic fatigue syndrome/myalgic encephalomyelitis (CFS/ME), irritable bowel syndrome (IBS), and fibromyalgia syndrome (FMS) –, and on somatic symptom disorders (SSD) (focusing on individuals with syndromes of multiple medically unexplained symptoms [MUS] or the precedingly used diagnostic label of somatoform disorders and classificatory equivalents). The complete search strategy is available in the Supplementary Material 1 or from PROSPERO 2017 CRD42017053596^1^1https://www.crd.york.ac.uk/PROSPEROFILES/53596_STRATEGY_20170105.pdf.

### Data Extraction

Titles, abstracts, and full texts of studies retrieved using the search strategy were entered at the Covidence platform^2^2https://www.covidence.org. Subsequently, the title and abstract of each retrieved reference were screened online by two review team members independently to identify reviews that met the eligibility criteria. Next, the full text of potentially eligible reviews was independently further assessed for inclusion by two review team members. Two raters tried to solve disagreements by finding consensus, if necessary, by involving a third review author.

A standardized, pilot tested form was entered at the REDCap-platform (Harris et al., 2009), hosted at Aarhus University and Utah State University, and was used to extract data from the finally included reviews (Box 1, for details, see Prospero protocol^3^3See Footnote 1.). For each eligible review, two review authors extracted data independently, discrepancies were identified and resolved through consensus discussion, with a third author where necessary. We did not allow raters to extract data and score quality of reviews they had authored.

Box 1 **Data That Were Extracted by Two Independent Researchers From Each Review**

- Type of review: narrative vs. systematic with/without meta-analysis
- Diagnostic concept: broad diagnostic concept vs. specific diagnosis
- Diagnoses covered: IBS, FMS, CFS/ME, SSD, others
- Number of included studies
- Minimum and maximum number of participants in the included studies
- Type of sample: clinical vs. population-based
- Aetiological domains covered: biological, psychological, social, health care system
- Main finding, and magnitude of main finding(s) for meta-analyses
- Authors' interpretation of the main findings
- Methodological quality, based on AMSTAR-2

### Methodological Quality

The methodological quality of each included review was assessed using the Assessment of Multiple SysTemAtic Reviews (AMSTAR) (Shea et al., 2007). AMSTAR was developed for critically appraising systematic reviews of randomised clinical trials. We adjusted the tool for our purposes as described below. According to the instructions of AMSTAR-2 (Shea et al., 2017), critical domains of the quality of reviews have to be identified. For this purpose three authors (MK, CR, and JR) independently indicated which of the 11 items of the original AMSTAR tool (Shea et al., 2007) indicated critical flaws, with discrepancies solved by consensus. The resulting unanimous critical items were Item 3 (Was a comprehensive literature search performed?), Item 6 (Were the characteristics of the included studies provided?), and Item 8 (Was the scientific quality of the included studies used appropriately in formulating conclusions?). Overall confidence in the results of the review was rated according to the AMSTAR-2 guidance as high (zero or one non-critical weakness), moderate (more than one non-critical weakness but no critical flaws), low (one critical flaw with or without non-critical weaknesses), or critically low (more than one critical flaw with or without non-critical weaknesses).

### Data Synthesis and Analysis

First, we obtained descriptive statistics of the frequency of diagnoses studied in the included reviews (FMS, IBS, CFS/ME, SSD), and of the type of review (narrative, systematic, meta-analysis) according to the year of publication. We distinguished between reviews that were diagnosis-specific, i.e., explored only one FSS diagnosis, and reviews that were based on a broad diagnostic concept, i.e., investigated at least two FSS simultaneously, SFD, SSD, and classificatory equivalents. Reviews that, for instance, investigated both FMS and other pain syndromes simultaneously were regarded broad reviews, but only data on FMS were extracted. Second, we analysed the predominant aetiological approach per diagnostic category, i.e., the frequency with which each domain of aetiological factors (see Box 1) was addressed. We defined reviews that assessed multiple aetiological factors from at least two aetiological domains simultaneously as those investigating a broad biopsychosocial model. Third, we analysed the frequency of specific aetiological factors per diagnostic category. Fourth, we assessed the methodological quality (high – moderate – low – critically low) for systematic reviews with or without meta-analysis per year of publication. Fifth, we provide a detailed overview of the few high-quality systematic reviews and analysed the associations of the investigated aetiological factors with FSS.

### Patient and Public Involvement

The central aim of our review was to systematically assess and analyse the variety and relative dominance of current aetiological views represented in systematic reviews of certain FSS. This research question did not provide opportunities to involve patients in the design, conduct, or reporting of our review. However, we plan to involve patients in disseminating our research findings (e.g., by presenting our results at meetings of patient interest and support groups).

## Results

### Search Results and Descriptive Variables of Included Reviews

We identified 5,605 reviews and assessed 980 full text articles for eligibility (see PRISMA checklist in the Supplementary Material 8 and PRISMA flow chart in Supplementary Material 10). We excluded 526 articles and included 454 articles (reporting on 452 reviews) in our descriptive analysis. Lists of all excluded and included reviews are in the online supplementary material (Supplementary Material 2 and 3).

Supplementary Material 4 provides characteristics of included systematic reviews with and without meta-analyses. Characteristics of included narrative reviews are summarised in Supplementary Material 5.

**Figure 1:**
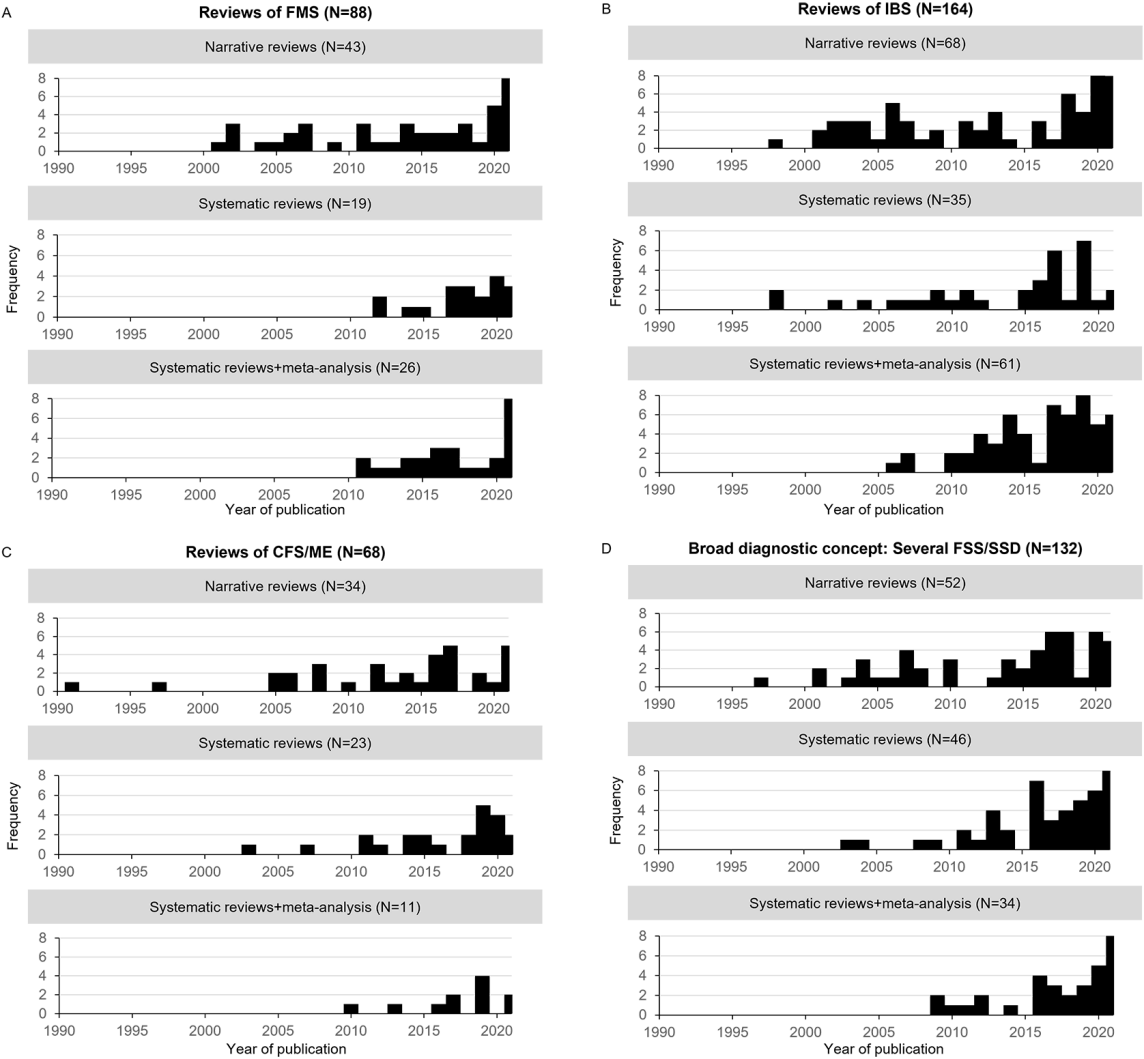
**A-D.** Frequency of Diagnosis-Specific (A-C) and Broad Reviews (i.e., Covering More Than One FSS Diagnosis and SSD) (D) Per Year of Publication Since 1990, Divided Into Narrative Reviews, Systematic Reviews Without and Systematic Reviews With Meta-Analyses *Note.* Reviews focusing on somatic symptom disorder are summarised under reviews with a broad diagnostic concept. FMS = fibromyalgia syndrome; FSS = functional somatic syndrome; IBS = irritable bowel syndrome; CFS / ME = chronic fatigue syndrome / myalgic encephalomyelitis; SSD = somatic symptom disorder.

Figure 1 provides the frequency of diagnosis-specific reviews (A-C) and reviews with a broader diagnostic conceptualisation (D) per publication year since 1990, divided into narrative reviews (*k* = 197), systematic reviews without meta-analyses (*k* = 123), and with meta-analyses (*k* = 132). The majority of reviews (71%, 320/452) were diagnosis-specific and of these 51% (164/320) were done in IBS. Focusing on systematic reviews with meta-analyses only, we found the same tendency: 74% (98/132) were diagnosis-specific and of these 62% (61/98) were IBS-specific. While the numbers of reviews on FMS and CFS/ME (from the pool of all included reviews) were comparable (88 and 68, respectively), the number of systematic reviews with meta-analyses on FMS (*k* = 26) was almost twofold compared with CFS/ME (*k* = 11) (Figure 1, A and C). We identified 17 reviews on SSD only, 3 narrative, 8 systematic without meta-analyses, and 6 systematic with meta-analyses. These are reviews summarised under reviews with a broader diagnostic concept in Figure 1D.

### Predominant Aetiological Approach in FMS, IBS, CFS/ME, and SSD

**Figure 2:**
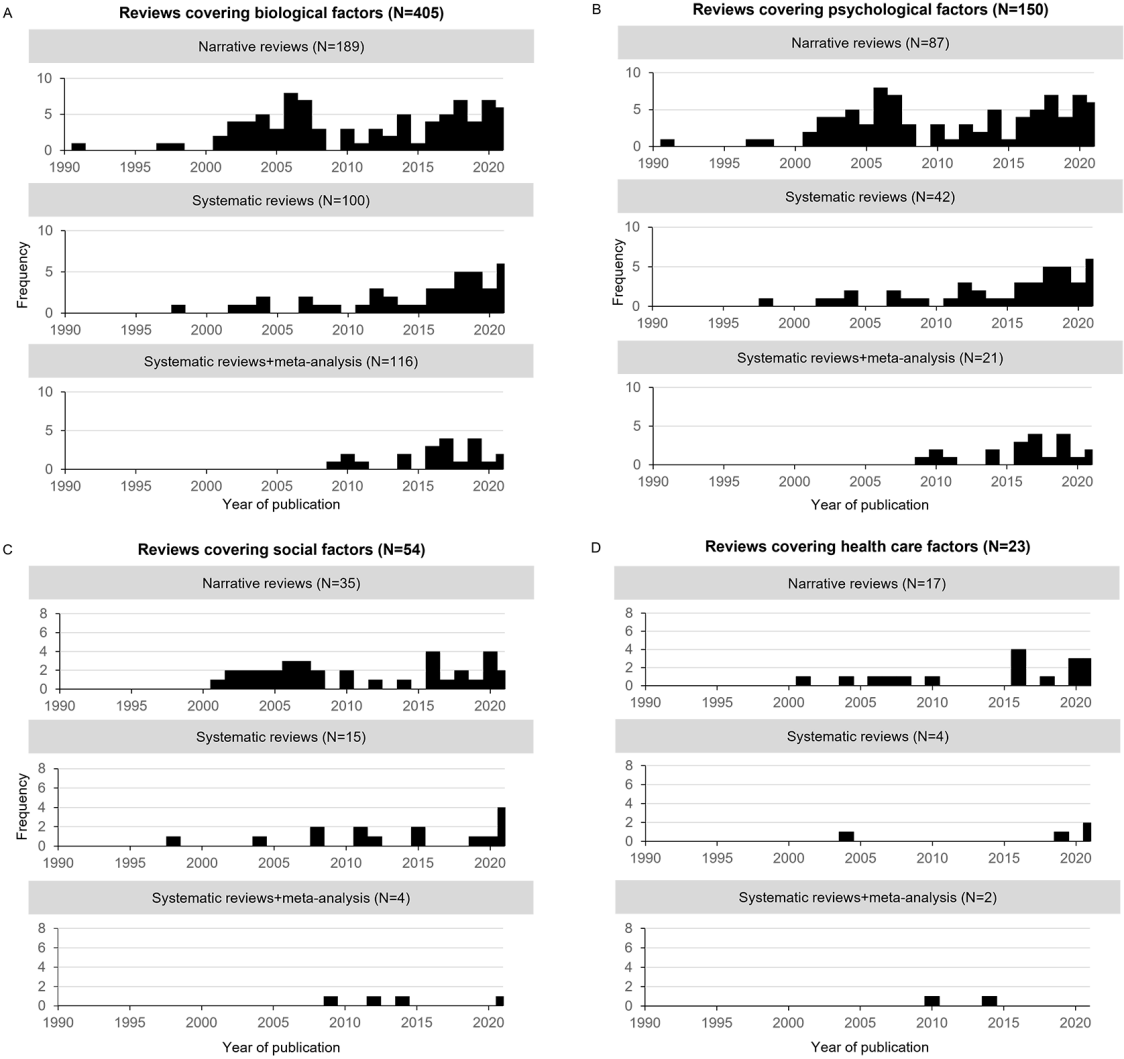
**A-D.** Frequency of Reviews (Regardless Investigated Diagnoses) Covering Biological (A), Psychological (B), Social (C) and Healthcare (D) Factors Per Year of Publication Since 1990, Divided Into Narrative Reviews, Systematic Reviews Without and Systematic Reviews With Meta-Analyses *Note.* One review may cover several factors, and hence appear in more than one of the Figures A-D.

Figure 2 provides the frequency of reviews covering biological (A), psychological (B), social (C), and healthcare (D) aetiological factors per publication year since 1990, divided into narrative reviews, systematic reviews with and without meta-analyses. In total 90% (405/452) of all reviews proposed or investigated biological factors to explain FSS, while 33% (150/452) proposed psychological, and 12% (54/452) social factors (Figure 2A-C). Several reviews included more than one group of aetiological factors (28%, 127/452), i.e., investigated aetiology on different levels simultaneously. Healthcare factors were discussed in 5% of the reviews (23/452) only (Figure 2D). The primary scientific interest was also pronounced in systematic reviews with meta-analyses: 88% (116/132) investigated biological factors, while only 20% (27/132) explored psychological, social, or healthcare factors. There was no indication that this relative dominance of biologically oriented reviews and meta-analyses changed during the past 20 years. Supplementary Material 4 and 5 shows that only 19% (87/452) of the included reviews are published in journals that are categorised in the field of psychiatry or social sciences (e.g., psychology, behavioural sciences, multidisciplinary sciences, sport sciences, public/environmental/occupational health), whereas the remaining reviews were mostly published in medical journals (e.g., gastroenterology, neurology, rheumatology) or journals in biology and pharmacology.

**Figure 3:**
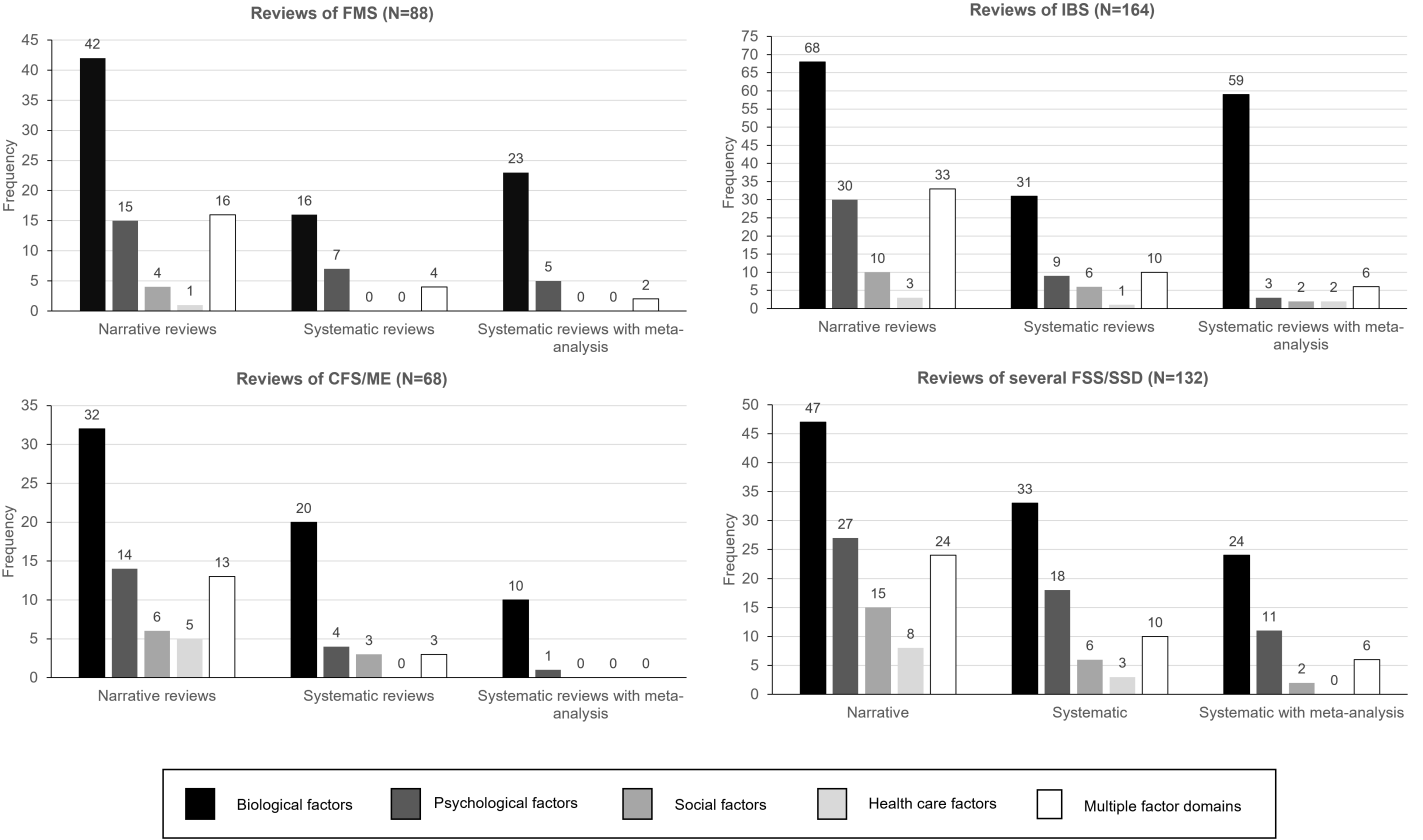
**A-D.** Aetiological Domains Covered in Diagnosis-Specific Reviews (A-C) and Reviews Applying a Broad Diagnostic Concept (D) *Note.* One review may propose or investigate more than one aetiological factor. 'Multiple factor domains' indicates reviews that include two or more domains simultaneously. FMS = fibromyalgia syndrome; FSS = functional somatic syndrome; IBS = irritable bowel syndrome; CFS / ME = chronic fatigue syndrome / myalgic encephalomyelitis; SSD = somatic symptom disorder.

Figure 3 provides the aetiological domains covered in diagnosis-specific reviews of FMS (A), IBS (B), CFS/ME (C) and reviews with broad diagnostic concepts (more than one FSS or SSD) (D). The dominance of a primarily biological approach (i.e., the attempt to describe the aetiology on a basic or “mechanistic” level only) was most evident in IBS-specific reviews: 96% (158/164) covered biological factors, while only 26% (42/164) covered psychological and 11% (18/164) social factors. A broad biopsychosocial model (i.e., acknowledging the interplay of aetiological factors) was proposed in 30% (49/164). The distribution of investigated domains of aetiological factors was similar in FMS, with 92% (81/88) addressing biological factors, 31% (27/88) psychological factors, 5% (4/88) social factors, and 25% (22/88) a broad biopsychosocial model. Regarding CFS/ME, in 28% (19/68) of the reviews addressed psychological factors, 13% (9/68) social factors, and broad models were included in 24% (16/68). However, the proportion of reviews investigating biological factors was with 91% (62/68) also high for CFS/ME.

**Figure 4:**
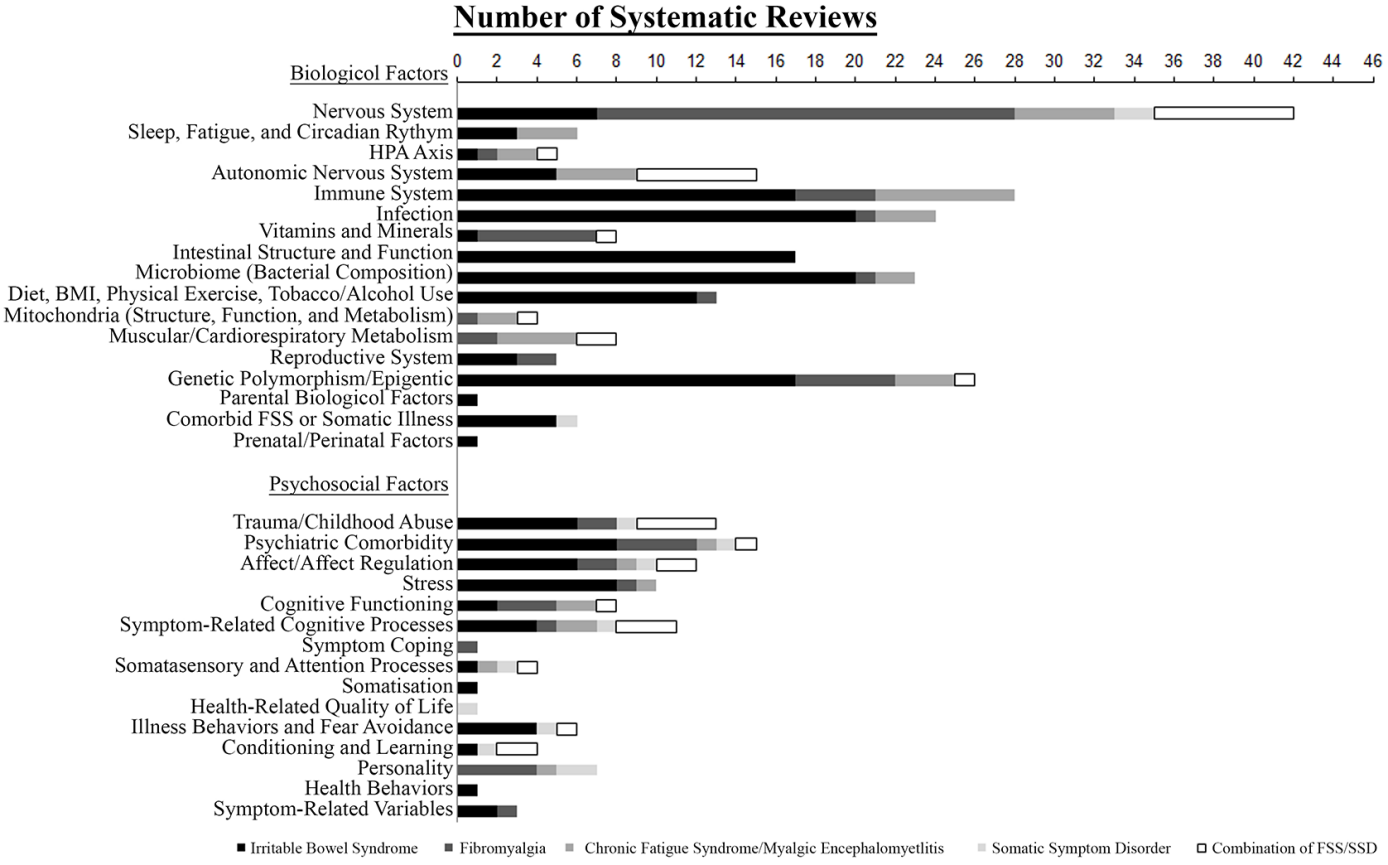
Frequency of Specific Investigated Biological and Psychosocial Factors in Systematic Reviews, Divided Into Reviews Addressing Irritable Bowel Syndrome, Fibromyalgia, Chronic Fatigue Syndrome, and Reviews Covering Somatic Symptom Disorder or More Than One of the Investigated Diagnoses Simultaneously

Figure 4 displays the number of systematic reviews (with and without meta-analyses) that investigated specific biological or psychosocial aetiological factors for each FSS diagnosis. Investigated biological factors included nervous and autonomic nervous system, sleep, hypothalamus pituitary adrenal (HPA)-axis, immune system, infection, vitamins and minerals, intestinal structure and function, intestinal bacterial composition, diet and body mass index (BMI), physical exercise, tobacco and alcohol use, mitochondrial structure or function and metabolism, muscular and cardiorespiratory metabolism, reproductive system, genetic polymorphisms and epigenetic changes, parental biological factors, comorbid FSS or somatic illness, and prenatal or perinatal factors (Supplementary Material 6A). Psychosocial factors also embraced a wide variety of aetiological theories, from developmental issues such as early trauma or impaired affect regulation, over learning processes such as attentional bias or conditioning, to specific illness behaviours or coping styles, and finally personality structure or interindividual (i.e., social or societal) factors (Supplementary Material 6B).

### Methodological Quality

The figures in Supplementary Material 10 provide the number of systematic reviews per publication year within the four quality strata. In general, the quality of reviews was low. Of the 255 included systematic reviews (with and without meta-analyses), the quality of 35 (14%) was critically low, 69 (27%) was low, 126 (49%) was moderate, and only 25 (10%) were considered of high quality. Figures A-D in Supplementary Material 9 also display the median year of publication within each quality category, ranging from 2015 for reviews of critically low quality to 2019 for reviews of moderate quality. The median publication year of the 25 high quality reviews was 2019 (range 2007-2021). Of these, 12 were IBS-specific, 3 FMS-specific, 2 CFS/ME-specific and the remaining 8 reviews were on individuals with multiple FSS.

### Currently Well-Documented Aetiological Factors

The table in the Supplementary Material 7 provides a detailed overview of the content and findings of the 25 systematic reviews of high quality. Moderate associations of *previous gastrointestinal infections* with IBS were reported in six reviews (Klem et al., 2017; Li et al., 2020; Saha et al., 2022; Schwille-Kiuntke et al., 2015; Svendsen et al., 2019; Wang et al., 2023). Limitations of these findings are high heterogeneity (Klem et al., 2017), and potential publication bias (Schwille-Kiuntke et al., 2015). Moreover, only a low number of reviews reported on specific pathogens and the results of the synthesis of data from these few reviews has to be interpreted with caution (Svendsen et al., 2019). IBS was the most commonly examined FSS regarding the previous infections as potential risk factor. There was only one study that examined relationship between a previous infection, a human herpes virus (HHV)-6 infection, and CFS (Mozhgani et al., 2022). The meta-analysis showed a 1.7 times increased risk in individuals with CFS to have a previous HHV-6 infection compared to healthy controls. *Lactose intolerance*, but not lactose maldigestion, was identified as risk factor of IBS in one review (Varjú et al., 2019). Differential analyses for IBS subtypes were done only in a small number of included studies (Varjú et al., 2019). Thus, conclusions of which subgroups are mostly affected by lactose intolerance cannot be drawn. Included studies vary substantially regarding the diagnostic criteria of IBS and diagnostic threshold of the lactose intolerance test (Varjú et al., 2019). *Gastrointestinal dysbiosis*, measured as count of lactobacillus, bifidobacterium, E coli, and enterobacter, significantly deviated between individuals with IBS and healthy control subjects (Wang et al., 2020). Here again, the increased heterogeneity of the studies included to the review limits the interpretability of the findings. Apart from lower *vitamin E levels*, no associations were found between vitamins or minerals and CFS or FMS (Joustra et al., 2017). The role of genetic factors, functional polymorphism in the gene encoding for activity of the *serotonin transporter protein* (SERT-P), was examined in individuals with IBS in two reviews (Van Kerkhoven et al., 2007; Zhu et al., 2018). Results are mixed, one analysis showed an increased risk of IBS associated with a functional polymorphism in the SERT-P gene (Zhu et al., 2018), whereas the other review showed no association (Van Kerkhoven et al., 2007). Two recent meta-analyses found low to moderate associations for *parasympathetic nervous system activity* as measured by means of high-frequency heart-rate variability (HRV) for IBS and FMS (Sadowski et al., 2021; Tracy et al., 2016). However, these estimates are based on only three to four studies per diagnosis, including a double publication on the same sample for FMS (Sadowski et al., 2021; Tracy et al., 2016). Additionally, one meta-analysis found changes in parasympathetic activity/HRV in a mixed group of CFS, FMS, and IBS patients, although these differences disappeared after correction for publication bias (Tak et al., 2009). A weak association between hypocortisolism (i.e. *HPA dysfunction*) and CFS/ME was found (Tak et al., 2011). One review examined the *immune status* in individuals with FMS (Andrés-Rodríguez et al., 2020). Compared to healthy controls, effect sizes indicated an increased level of different types of interleukins in subjects who are diagnosed with FMS. Another review showed a significantly decreased level of *conditioned pain modulation* in individuals with IBS compared to a healthy control sample (Albusoda et al., 2018). Amiri et al. (2021) demonstrated a significantly lower *nociceptive flexion reflex* threshold in patients with FMS. The nociceptive flexion reflex is a physiological, polysynaptic reflex triggered by a nociceptive stimulus activating a withdrawal response (Smith et al., 2017). A decreased nociceptive flexion reflex threshold has been discussed as a possible biomarker of central sensitization that may cause alteration of central nervous system processing in individuals with chronic musculoskeletal-related pain condition (Smith et al., 2017). Núñez-Fuentes et al. (2021) examined in their meta-analysis an association between alterations in *postural balance* and FMS. The authors demonstrated large effects indicating that patients with FMS show significantly worse scores on a variety of different measures of postural balance compared to healthy controls.

The following psychological variables were examined in high-quality reviews: history of sexual abuse and pain catastrophising. Moderate associations were reported for a *history of sexual abuse* and a lifetime diagnosis of IBS (Paras et al., 2009). The association of sexual abuse with FMS is less straightforward; it was only significant in a sensitivity analysis that was restricted to severe abuse, specifically rape (Paras et al., 2009). Limitations include unexplained heterogeneity, methodological limitations, recall bias, and the unknown generalisability to men, since studies were mainly performed in women. Finally, one review showed that *pain catastrophising* explains to a moderate extend variance of pain intensity and disability in individuals with a combination of FMS and CFS/ME (Martinez-Calderon et al., 2019).

## Discussion

### Statement of Principal Findings

This systematic umbrella review assessed the variety and relative dominance of aetiological factors in both narrative and systematic reviews of the three most acknowledged FSS and SSD (focusing on individuals with syndromes of multiple medically unexplained symptoms [MUS] or the precedingly used diagnostic label of somatoform disorders and classificatory equivalents). Although the number of systematic reviews has been increasing substantially in recent years, the review quality has only marginally improved. Almost three-quarter of the reviews was diagnosis-specific, with IBS being the most prominent syndrome. Very few reviews have taken a broad view across diagnoses. This is remarkable given the substantial diagnostic overlap among syndromes. This means that most individuals who are included in a study of IBS, for example, may suffer from other, co-morbid FSS – however, very few original studies on IBS distinguish between study participants who have IBS only, and those who have IBS with concomitant other FSS. Results of a recently published systematic review of cohort studies on predictors of the onset of persistent somatic symptoms confirm a similar focus on irritable bowel syndrome (Kitselaar et al., 2023).

It is important to note that the overlap between the medical and psychiatric diagnoses has been reduced in the most recent version of DSM (American Psychiatric Association, 2013). SFD were included in DSM-III (American Psychiatric Association, 1980) and DSM-IV (American Psychiatric Association, 2000), and these diagnoses were based on the presence of somatic symptoms for which there were no demonstrable organic findings or known physiologic mechanisms. DSM-5 (American Psychiatric Association, 2013) replaced this category, given that it was not considered appropriate to make a mental disorder diagnosis solely because a medical cause of the somatic symptoms cannot be demonstrated. The new diagnosis of SSD is made based on the presence of somatic symptoms, explained or unexplained, in combination with dysfunctional cognitions, emotions, or behaviours. This implies that the overlap in diagnostic criteria between FSS and SSD is largely reduced.

Our umbrella review clearly showed a predominance of the biological perspective: Biological factors were included in 90% of reviews, whereas 33% discussed psychological factors; only 28% discussed two or more domains of aetiological factors. Only 5% and 12% discussed healthcare or societal factors, respectively. This biological predominance, i.e. interest on the most basic aetiological level seems to be common and has also been demonstrated in other recently published systematic reviews, for example a review on cohort studies on predictors of the onset of persistent somatic symptoms (Kitselaar et al., 2023). It is in contrast with the views of many health care professionals and with current prevailing clinical management strategies that focus on doctor-patient communication, patients’ illness perceptions and illness behaviours, and other healthcare and psychosocial factors (Henningsen et al., 2007; Henningsen, Zipfel, et al., 2018). In other words, the predominant management strategies are not backed up by firm aetiological research. This discrepancy between theoretical assumptions guiding clinical practice and those directing the dominant research focus seems to rely on fundamentally different views on causality and explanatory mechanisms, thereby contributing to enduring controversy and heated debates about legitimisation and “epistemic justice” to patients with FSS (Bernstein, 2016; Cohen, 2017; Mikocka-Walus et al., 2016; Spandler & Allen, 2018).

Specific aetiological factors included both previous or trait factors as well as current or state factors. While predisposing or triggering risk factors may help to identify people at risk or to prevent the development of FSS through control or even elimination of such factors, current or perpetuating factors may be of special interest, as these are potentially modifiable and therefore may be targets for intervention. The few high-quality reviews suggest that both biological (e.g., infection) and psychosocial (i.e., history of sexual abuse or pain catastrophising) factors can increase the risk of FSS. This is in line with current illness models for FSS (e.g., Deary et al., 2007). Four high quality reviews suggest involvement of the ANS in painful FSS (Sadowski et al., 2021; Tak et al., 2009; Tracy et al., 2016) in painful FSS and syndromes of fatigue and exhaustion (Tak et al., 2011). These findings suggest that different symptom clusters may be associated with specific pathophysiological pathways, while a more general dysfunction in interoception may be generic and of relevance for all FSS (Henningsen, Gündel, et al., 2018).

In summary, these high quality reviews show that there is a multiplicity of factors associated with FSS. This could be interpreted as an indicator of subgroups in a group of people diagnosed with a particular syndrome, who have different aetiological pathways. A recently published study (Kendler et al., 2022) examined genetic risk patterns in FSS such as IBS, CFS, and FMS as well as in a prototypic mental health condition, such as depression, and a prototypic somatic condition, rheumathoid arthritis. The authors could demonstrate unique profiles of family genetic risk scores in individuals with specific single FSS that were very different to major depression and rheumatoid arthritis. Another recently published study (Creed, 2022b) examined risk factors in individuals with self-reported IBS. This study identified partly overlapping and partly unique patterns of risk factors for a subgroup of IBS patients with previous mental health conditions compared to individuals with IBS but no previous mental health problem.

There is another important aspect that we have to consider when we interpret the high-quality reviews included in our umbrella review: We observe a relative infrequency of studies that measure several putative risk factors simultaneously. There are some single examples, such as the rather “biological“ study of IBS by Dunlop et al. (2003) which examined risk factors simultaneously. Dunlop et al. (2003) found that both increased enterochromaffin cell counts and depression were equally important predictors of developing post-infectional IBS. However most studies of biological factors in our umbrella review fail to include a psychological or social measure in addition to the biological one. Another example concerned fibromyalgia, for which numerous somatic symptoms are a risk factor (Creed, 2022a). However, a study by Creed (2022a) demonstrated that there are new onset cases of FMS with only few somatic symptoms and that this subgroup of FMS patients shows, compared to individuals with FMS and numerous other somatic symptoms, a unique pattern of risk factors. These results are in accordance with Kendler’s concerns that we finally have to withdraw from the dualistic or dichotomous thinking within psychiatry and have to acknowledge that biological, psychological, and social cultural domains are inter-twined with each other in aetiological pathways (Kendler, 2012).

### Strength and Weaknesses of the Review

Our review has a few limitations. First, we only included the most common FSS. Other intensively investigated syndromes or symptoms, such as chronic low back pain, might have added other well-documented aetiological factors (Vlaeyen et al., 2018). Second, systematic reviews of chronic pain not always provided specific results for primary pain as opposed to mixed pain, or secondary pain, and it was often difficult to extract specific details for FMS and IBS from those reviews. Third, our quality rating was done using a tool that was constructed for the evaluation of reviews of intervention trials. Fourth, we only included reviews, meaning that the most novel aetiological factors as investigated in empirical studies may not have been covered. Finally, the reviews included in our umbrella review are mainly based on studies implementing cross-sectional designs. We did not include animal research that would allow experimental designs and conclusions about causal factors.

Our review has also important strengths. First, it is the first comprehensive overview that covers aetiological factors of the most well-known FSS together. Second, both medical and psychiatric definitions of these syndromes were used, thereby avoiding bias. Third, we restricted our analysis to reviews which are typically the primary sources for guidelines that affect daily clinical practice. Finally, we were interested in aetiological factors on various levels. Therefore, we regard this review a very first step to unravel the "dappled nature of causes" of these syndromes (Kendler, 2012).

### Implications for Research and Clinical Management

Our results have important implications for future research: First, we showed that the FSS are largely studied separately, with only a minority of reviews including more than one syndrome, despite the empirical overlap in symptoms and shared non-symptom characteristics of the patients (Wessely et al., 1999) suggesting that they constitute a family of disorders (Fink & Schröder, 2010; Janssens et al., 2015). Future studies should investigate them together, since this could facilitate the identification of both syndrome-specific and generic aetiological and pathogenic factors at different levels, which would critically inform the discussion between “splitters” and “lumpers” (Fink, 2017).

Second, current explanatory models promote a biopsychosocial approach to diseases in general (Rief & Broadbent, 2007; Witthöft & Hiller, 2010). However, since the appearance of IBS, FMS and CFS/ME as MESH terms in MEDLINE from 1989 onwards, very few systematic reviews have used comprehensive disease models to guide the selection of aetiological factors in FSS research. The still widely acknowledged dualistic "hardware versus software" rationale likely has slowed down scientific progress and might continue to do so (Fink & Schröder, 2010; Rosmalen, 2010) until it is replaced by empirically based pluralism (Kendler, 2012). It would be a major step forward if different groups of aetiological factors on different levels would be combined into one longitudinal, multidisciplinary study, in order to examine their interrelations (Rosmalen, 2010). This is currently done in a number of large epidemiological studies, e.g., DanFunD (Dantoft et al., 2017) and LifeLines (Scholtens et al., 2015).

Our review also has important implications for clinical management: The knowledge about aetiological factors that has been gained from our review has to be translated into explanatory models for single patients. For each individual case the contribution of biological, psychosocial, and healthcare factors has to be weighted, acknowledged, and negotiated with the patient. After all, it is the individual patient’s history of risk and protective factors as well as his/her/their needs and wishes that is the foundation on which personalised care is built, not the theoretical preferences of the clinician (Gask, 2018).

### Conclusions and Future Research

In summary, our umbrella review reveals that the literature on aetiological factors in FSS and SSD is predominantly characterised by a diagnosis-specific perspective with a focus on biological factors, based on a purely biomedical conceptualisation of FMS, IBS and CFS/ME as distinct disease entities. SSD, or the previously used diagnostic category somatoform disorders, is only sparsely investigated. The majority of reviews provide expert views rather than firm results, and overall the reviews are very often of low quality and mostly implement only cross-sectional designs. We however identified 25 systematic reviews, partly including meta-analyses, that provide information of a variety of biological factors and some psychological factors that function as potential mechanisms. The information gained from these high-quality studies should be translated into explanatory models for patients.

We believe that future research should strive for higher scientific standards and more interdisciplinary research collaboration. We recommend that more research work should focus on examining the different FSS together. Examining differences as well as similarities of specific FSS could be reached by an approach that uses the same data set gained in the same population (Monden et al., 2022). Recognising these health conditions as closely related and including all relevant factors that potentially play a role may lead to distinguishing evidence-based subtypes or syndromes that may benefit from person-centred approaches. It is our hope that this review contributes to the development of a commonly accepted and evidence-based conceptualisation of FSS and SSD in both medicine and psychiatry.

## Supplementary Materials

The Supplementary Materials (Kleinstäuber et al., 2023) contain the following items:

- **Supplementary Material 1.** Results of the search of electronic literature database
- **Supplementary Material 2.** References of included reviews (*k* = 452)
- **Supplementary Material 3.** References of reviews excluded after fulltext review (*k* = 526)
- **Supplementary Material 4.** Characteristics of included systematic reviews without meta-analysis (*k* = 123) and systematic reviews with meta-analyses (*k* = 132)
- **Supplementary Material 5.** Characteristics of included narrative reviews (*k* = 197)
- **Supplementary Material 6A.** Specific biological factors investigated in diagnosis-specific systematic reviews (FMS, IBS, CFS/ME and SSD) or systematic reviews that investigated at least two of these diagnoses simultaneously (combinations of FSS/SSD)
- **Supplementary Material 6B.** Specific psychosocial factors investigated in diagnosis-specific systematic reviews (FMS, IBS, CFS/ME and SSD) or systematic reviews that investigated at least two of these diagnoses simultaneously (combinations of FSS/SSD)
- **Supplementary Material 7.** Characteristics of systematic reviews with meta-analysis with an overall rating ‘high’ of confidence in the results of the review according to AMSTAR-2 (*k* = 25)
- **Supplementary Material 8.** PRISMA Checklist
- **Supplementary Material 9.** Study selection process (PRISMA flow chart)
- **Supplementary Material 10.** Frequency of systematic reviews per year of publication since 1990, divided into reviews of critically low, low, moderate and high quality, according to the Assessment of Multiple SysTemAtic Reviews (AMSTAR-2). The grey vertical lines indicate the median (Md) publication year within each quality stratum.

KleinstäuberM.
SchröderA.
DaehlerS.
PallesenK. J.
RaskC. U.
SanyerM.
Van den BerghO.
Weinreich PetersenM.
RosmalenJ. G. M.
 (2023). Supplementary materials to "Aetiological understanding of fibromyalgia, irritable bowel syndrome, chronic fatigue syndrome and classificatory analogues: A systematic umbrella review"
[Additional information]. PsychOpen. 10.23668/psycharchives.13273
PMC1086363738356902

## Data Availability

Data are available on reasonable request from the corresponding author.
